# Broadband High Optical Transparent Intelligent Metasurface for Adaptive Electromagnetic Wave Manipulation

**DOI:** 10.34133/research.0334

**Published:** 2024-03-11

**Authors:** Chao Xia, Zhengang Lu, Yilei Zhang, Jiubin Tan

**Affiliations:** ^1^ Ultra-Precision Optical & Electronic Instrument Engineering Center, Harbin Institute of Technology, Harbin 150001, China.; ^2^ Key Lab of Ultra-precision Intelligent Instrumentation (Harbin Institute of Technology), Ministry of Industry and Information Technology, Harbin 150001, China.

## Abstract

Intelligent metasurfaces have garnered widespread attention owing to their properties of sensing electromagnetic (EM) environments and multifunctional adaptive EM wave manipulation. However, intelligent metasurfaces with broadband high optical transparency have not been studied to date, and most of the previous intelligent metasurfaces lack an integrated design for their actuators and sensors, resulting in lower integration levels. This study proposes a novel intelligent metasurface with adaptive EM wave manipulation ability and high optical transparency from visible to infrared bands. This metasurface consists of a transparent and current-controlled reconfigurable metasurface as an actuator by integrating patterned vanadium dioxide (VO_2_) into metal-meshed resonant units, transparent broadband microstrip antenna as a sensor, recognition-and-feedback module, and actuator- and sensor-integrated design on the same substrate. The EM-regulating capability of the designed transparent intelligent metasurface is theoretically analyzed using the coupled mode theory, and a prototype metasurface device is fabricated for experimental verification. Simulation and experimental results demonstrate that the metasurface exhibits over 80% normalized transmittance from 380 to 5,000 nm and adaptive EM wave manipulation (reflective strong shielding function with a shielding efficiency of over 24 dB, high transmittance function with a transmission loss of 1.24 dB, and strong absorption function with an absorption of 97%) according to the EM wave power parameters without manual intervention. This study provides an avenue for transparent intelligent metasurfaces with extensive application prospects in areas such as intelligent optical windows, radar enclosures, and communication.

## Introduction

In recent years, intelligent metasurfaces have shown tremendous potential for applications such as intelligent communication and adaptive camouflage owing to their ability to adaptively manipulate electromagnetic (EM) waves without human intervention [1,2]. With the rapid development of unmanned systems, artificial intelligence, and the construction and application of advanced intelligent equipment or systems, enhancing the EM manipulation capability of equipment has become a crucial challenge for improving the adaptability of various intelligent equipment to EM environments and ensuring their safe and reliable operation. In particular, the metasurface used as a window needs to adaptively manipulate the EM transmission function according to the changes in the EM environment and achieve high optical transparency from the visible to the infrared band in the applications of intelligent equipment that require visual observation or infrared detection, such as autonomous vehicles, aerospace equipment windows, smart military equipment, and communication detection windows. However, to the best of our knowledge, intelligent metasurfaces with high optical transparency in the visible–infrared band have not yet been reported.

To date, several intelligent metasurfaces with different functions have been reported, such as adaptive camouflage [3,4], adaptive EM function switching [5–7], adaptive beam control [8–12], and intelligent communications [13,14]. However, the crucial tunable devices for these intelligent metasurfaces are lumped elements that typically require printed circuit boards and surface-mounting techniques to solder the lumped elements to the metasurfaces, such as positive-intrinsic-negative or varactor diodes. Furthermore, some elements even require drilling holes to build complex feed networks. Therefore, intelligent metasurfaces with lumped elements are difficult to use in optically transparent areas. In addition, the majority of intelligent metasurfaces feature separate designs for their actuators and sensors, with the sensors often being large external components that are independent of the actuators, such as antennas and cameras. Consequently, this results in very low integration levels for intelligent metasurfaces, significantly affecting their use in practical applications.

The selection of tunable materials is crucial for intelligent metasurfaces. Currently, there are many active tunable materials available, such as graphene [15,16], semiconductor [17], ferroelectrics [18], liquid crystals [19,20], and phase change materials [21–24]. Vanadium dioxide (VO_2_) is considered to be one of the most promising application-tunable phase change materials because of its ability to realize a drastic insulator–metal phase transition (in sheet resistance with theoretically 5 orders of magnitude) at approximately 68 °C (the closest room-temperature transition temperature among phase change materials). In addition, VO_2_ exhibit a variety of transition-triggering mechanisms, such as external Joule heat and electric, magnetic, stress, and light fields [25,26]. In addition, VO_2_ can be conveniently integrated into metasurfaces [27].

In this study, a novel intelligent metasurface is demonstrated with broadband high optical transparency and adaptive EM wave manipulation ability. A transparent and current-controlled reconfigurable metasurface is realized as an actuator through the integrated design of patterned VO_2_ and metal-meshed resonant units. The integration of the sensing and actuator is realized by integrating the transparent reconfigurable metasurface with the transparent broadband microstrip antenna on the same substrate. The external signal processing and feedback modules are designed to build a closed-loop system without manual intervention. The designed transparent intelligent metasurface senses the power of the environmental EM wave through the integrated transparent broadband microstrip antenna, power detector, and field-programmable gate array (FPGA). Subsequently, the upper computer sends out the corresponding feedback control commands according to different EM environments. Finally, the corresponding reflective strong shielding, high transmittance, and strong absorption functions are switched through the feedback output current. Simultaneously, the metasurface realizes broadband high optical transparency using micrometer-scale patterned VO_2_ integrated with the metal-meshed resonant unit. The EM-regulating capability of the designed transparent intelligent metasurface is theoretically analyzed using the coupled mode theory (CMT), and a prototype metasurface device is fabricated for experimental verification. This study provides a feasible method for the realization of optically transparent intelligent metasurfaces.

## Results

### Architecture and simulation of the optically transparent intelligent metasurface

**Fig. 1.**
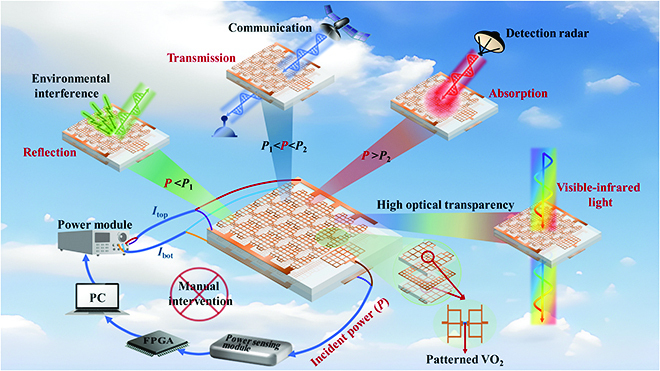
Schematic of the optically transparent intelligent metasurface. *P* is the incident wave power, *P*_1_ and *P*_2_ are the power threshold, and *I*_top_ and *I*_bot_ are the control currents of the top and bottom layers respectively. The metasurface adaptively reflects environmental interference EM waves (*P < P*_1_), transmits own communication EM waves (*P*_1_ < *P < P*_2_), and absorbs detection radar waves (*P > P*_2_) and the whole process without manual intervention. In particular, the metasurface can achieve high transmittance from visible to infrared bands.

A schematic diagram of the proposed transparent intelligent metasurface system consisting of an actuator, sensor, power sensing module, and feedback control module is illustrated in Fig. 1. The actuator is a transparent and current-controlled reconfigurable metasurface integrated with patterned VO_2_. The sensor is a transparent broadband microstrip antenna. The transparent broadband microstrip antenna and reconfigurable metasurface are designed on the same substrate to integrate sensing and actuation. The power sensing module includes a low-noise power amplifier (LNA), power detector, and analog-to-digital converter (ADC). The feedback control module consists of an FPGA, a computer for upper-level control, and a power module.

The proposed transparent intelligent metasurface receives and measures the power of incident EM waves using the sensor and power sensing module. The required bias currents are immediately provided to the transparent reconfigurable metasurface after computation and decision making in the adaptive feedback control module, enabling it to automatically switch to the corresponding EM function. Specifically, the designed transparent reconfigurable metasurface utilizes current to induce the phase transition of VO_2_ and generate 3 different EM functionalities: reflective strong shielding, high transmission, and strong absorption. When the power of incident EM waves is very low (<*P*_1_), it is considered as environmental interference wave. In this case, the transparent reconfigurable metasurface acts as a reflection shielding state to avoid interference damage to internal electronic devices. When the power of the incident EM wave increases to the communication threshold (*P*_1_~*P*_2_), it is considered as own communication EM waves, and the transparent reconfigurable metasurface is controlled to switch to the high transmission state. However, when the power of the incident waves continues to increase (>*P*_2_), it is considered as a threat from detection radar waves, and the transparent reconfigurable metasurface is controlled to switch to a strong absorption state, thereby achieving radar stealth capability. Additionally, the proposed metasurface achieves high visible–infrared broadband transmittance by using micrometer-scale patterned VO_2_ instead of traditional opaque lumped components, and low-duty metal meshes instead of metal structural parts.

**Fig. 2.**
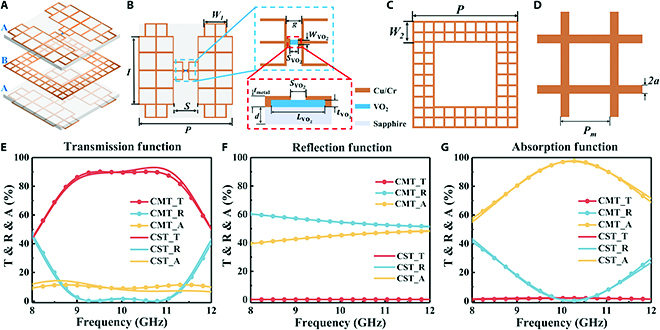
(A) Unit structure of the transparent reconfigurable metasurface. (B) A layer structure of the transparent reconfigurable metasurface unit, where the inset includes an enlarged view of the slit integrated with patterned VO_2_ and sketch of the section. (C) B layer structure of the transparent reconfigurable metasurface unit. (D) Square metal mesh used in meshed reconfigurable metasurface. (E) Transmission function. (F) Reflection function. (G) Absorption function.

The transparent reconfigurable metasurface consists of an ABA 3-layer structure, where the 2 A layers have identical structures, as shown in Fig. 2A. Schematic illustrations of the A and B layers are shown in Fig. 2B and C, respectively. Layer A consists of 2 mirrored metal patches that are connected through a rectangular-patterned VO_2_ patch. Layer B consists of square ring-shaped metal patches. All the metal parts of the ABA structure are meshed using square metal meshes to achieve broadband transparency, as shown in Fig. 2D. The structural parameters are *P* = 2.4 mm, *I* = 1.6 mm, *S* = 0.3 mm, *W*_1_= 0.7 mm, *g* = 36 μm, *S*_VO_2__ = 5 μm, *W*_VO_2__ = 15 μm, *L*_VO_2__ = 30 μm, *t*_VO_2__ = 250 nm, *t*_metal_ =0.4 μm, *d* = 1 mm, *W*_2_ = 0.36 mm, *P_m_* = 100 μm, and 2*a* = 6 μm. The phase transition state of the patterned VO_2_ is directly controlled by providing bias currents to the 2 A layers, thereby controlling the connection state of the mirrored meshed metal patches. Consequently, different EM responses of the metasurface can be achieved.

Finite integration simulation software (CST Microwave Studio) was used to simulate the Ku-band (8 to 12 GHz) and theoretically analyze the EM transmission characteristics of the designed transparent reconfigurable metasurface. The theoretical dielectric constant of the sapphire substrate was 9.4, and the patterned VO_2_ was modeled as a variable sheet resistance film with a range of 200 to 400,000 Ω/sq. The simulation results are represented as solid lines in Fig. 2E to G. The metasurface exhibited high transmittance function when the patterned VO_2_ in both A layers are insulating state (the sheet resistance in the top layer *R*_*sq*_*t*_ = 400,000 Ω/sq and that in the bottom layer *R*_*sq*_*b*_ = 400,000 Ω/sq). Here, the transmittance is greater than 70% from 8.55 to 11.62 GHz and reaches 93% at 10.72 GHz. The reflectance of the metasurface is greater than 50% and transmittance is almost zero in the range of 8 to 12 GHz when patterned VO_2_ in the 2 A layers are in a metallic state (*R*_*sq*_*t*_ = *R*_*sq*_*b*_ = 200 Ω/sq). Here, the metasurface primarily exhibited the reflective EM shielding function. The highest absorption reached more than 97% at 10.21 GHz when the top and bottom patterned VO_2_ materials are in intermediate phase transition and metallic states (*R*_*sq*_*t*_ = 1,400 Ω/sq and *R*_*sq*_*b*_ = 200 Ω/sq), respectively. In addition, the absorption is greater than 55% and the transmittance was almost zero in the range of 8 to 12 GHz, verifying that the metasurface possessed EM stealth functionality based on the high absorption capacity.

CMT is also used for analysis to further understand the above simulation results for the proposed transparent reconfigurable metasurface. As shown in Fig. S1, the B layer of the proposed ABA-type metasurface exhibits EM shielding capability greater than 16 dB from 8 to 12 GHz, which can provide an opaque background for the entire ABA structure in the operating frequency band. The resonant structures of the 2 A layers contribute to 2 electric resonance modes individually. Thus, the whole ABA structure can be described as a 2-mode system within an opaque background. According to CMT theory, the time evolution equations of the amplitudes *a*_1_ and *a*_2_ of the resonant modes provided by the 2 A layers are as follows [28,29]:

$$\left\{\begin{array}{c} \frac{1}{2\pi }\frac{d}{\mathrm{dt}}\left(\begin{array}{c} a_{1}\\ a_{2} \end{array}\right)=j\left(\begin{array}{cc} f_{1} & \kappa \\ \kappa & f_{2} \end{array}\right)\left(\begin{array}{c} a_{1}\\ a_{2} \end{array}\right)+\left(\begin{array}{cc} -\Gamma_{1} & X\\ X & -\Gamma_{2} \end{array}\right)\left(\begin{array}{c} a_{1}\\ a_{2} \end{array}\right)+\left(\begin{array}{cc} -\Gamma_{1}^{A} & 0\\ 0 & -\Gamma_{2}^{A} \end{array}\right)\left(\begin{array}{c} a_{1}\\ a_{2} \end{array}\right)+\left(\begin{array}{cc} d_{11} & d_{21}\\ d_{12} & d_{22} \end{array}\right)\left(\begin{array}{c} S_{1}^{\text{In}}\\ S_{2}^{\text{In}} \end{array}\right)\\ \left(\begin{array}{c} S_{1}^{\text{Out}}\\ S_{2}^{\text{Out}} \end{array}\right)=\left(\begin{array}{cc} C_{11} & C_{12}\\ C_{21} & C_{22} \end{array}\right)\left(\begin{array}{c} S_{1}^{\text{In}}\\ S_{2}^{\text{In}} \end{array}\right)+\left(\begin{array}{cc} d_{11} & d_{12}\\ d_{21} & d_{22} \end{array}\right)\left(\begin{array}{c} a_{1}\\ a_{2} \end{array}\right) \end{array}\right.$$ (1)

where *f_i_* (*i* = 1, 2) represents the resonant frequency corresponding to the resonant mode provided by the 2 A layers, *κ* refers to the near-field coupling of the 2 modes on the B layer, *Χ* represents the far-field coupling of the 2 modes, *d_li_* (*l* = 1, 2) denotes the coupling between the *i*th mode and the *l*th external port. Γ*_i_* represents the radiation attenuation rate of the *i*th mode, and $\Gamma_{i}^{A}$ represents the absorption attenuation rate of the *i*th mode. $S_{i}^{\text{In}}$ and $S_{i}^{\text{Out}}$ respectively denote the incident and outgoing waves at the *i*th port. *C_mn_* represents the coupling coefficient from the *n*th port to the *m*th port. Based on the time reversibility and energy conservation, the following constraints exist:

$$\begin{array}{l} X=-\frac{\left(d_{11}^{*}d_{12}+d_{21}^{*}d_{22}\right)}{2}\\ \Gamma_{i}=\frac{{\left|d_{1i}\right|}^{2}+{\left|d_{2i}\right|}^{2}}{2}\\ \left(\begin{array}{cc} C_{11} & C_{12}\\ C_{21} & C_{22} \end{array}\right){\left(\begin{array}{cc} d_{11} & d_{21}\\ d_{12} & d_{22} \end{array}\right)}^{*}=-\left(\begin{array}{cc} d_{11} & d_{12}\\ d_{21} & d_{22} \end{array}\right) \end{array}$$ (2)

Due to the presence of an opaque background (B layer), we can obtain *C*_11_ = *C*_22_ =  − 1 and *C*_12_ = *C*_21_ = 0. Additionally, assuming that the original 2 resonant modes are identical, we have *d*_11_ = *d*_22_ = *d*, and *d*_12_ = *d*_21_ = 0. Therefore, by solving Eq. 1, we can ultimately obtain the transmission and reflection coefficients of the overall structure as follows:

$$\begin{array}{l} t=\frac{d^{2}}{2}\times \frac{\left(Q_{1}-Q_{2}\right)}{Q_{1}Q_{2}-P^{2}}\\ r=-1+\frac{-d^{2}P+\frac{d^{2}}{2}\left(Q_{1}+Q_{2}\right)}{Q_{1}Q_{2}-P^{2}} \end{array}$$ (3)

where $\begin{array}{c} Q_{1}=j\left(f-f_{1}+\kappa \right)+d^{2}/2+\left(\Gamma_{1}^{A}+\Gamma_{2}^{A}\right)/2 \end{array}$, $Q_{2}=$$\left(f-f_{2}+\kappa \right)+d^{2}/2+\left(\Gamma_{1}^{A}+\Gamma_{2}^{A}\right)/2$, and $P=\left(\Gamma_{1}^{A}-\Gamma_{2}^{A}\right)/2$. Then, we can get the absorption by the following formula: *A* = 1 − |*t*|^2^ − |*r*|^2^. Therefore, we can obtain 6 independent model parameters, which are *f*_1_, *f*_2_, $\;\Gamma_{1}^{A}$, $\;\Gamma_{2}^{A}$, *d*, and *κ*. *κ* and *d* are mainly determined by the original resonant structures (patterned VO_2_ sheet resistance to infinity), while *f*_1_, *f*_2_ and $\;\Gamma_{1}^{A}$, $\;\Gamma_{2}^{A}$ are mainly determined by the sheet resistance of patterned VO_2_. Since it is difficult to calculate these parameters quantitatively, we obtained the corresponding parameter values from fitting with CST simulation results. The CMT results are shown as scatter plots in Fig. 2E to G. The CMT fitting results in Fig. 2 indicated a high consistency between the fitting curves for different EM functionalities of the metasurface and CST simulation results, thereby demonstrating the correctness of the CMT model. The fitted values of the 6 independent CMT model parameters are presented in Table.

**Table.** CMT fitting parameters of different electromagnetic functions (the units of *f*_1_, *f*_2_, $\;\Gamma_{1}^{A}$, $\;\Gamma_{2}^{A}$, *d*, and *κ* are all GHz)

Different electromagnetic functions	*f*_1_, *f*_2_	$\Gamma_{1}^{A}$, $\;\Gamma_{2}^{A}$	*d*	*κ*
Transmission: *R*_*sq*_*t*_ = *R*_*sq*_*b*_ = 400*K*Ω/sq	10.06,10.06	0.07,0.07	1.59	−1.44
Reflection: *R*_*sq*_*t*_ = *R*_*sq*_*b*_ = 200Ω/sq	12.37,12.37	7.50,7.50	1.59	−1.44
Absorption: *R*_*sq*_*t*_ = 1,400Ω/sq, *R*_*sq*_*b*_ = 200Ω/sq	8.14,12.37	1.36,7.50	1.59	−1.44

It can be observed that in the reflection and transmission functionalities, the sheet resistances of the patterned VO_2_ in the 2 A layers are the same. In this case, we can consider that *f*_1_ = *f*_2_ and $\Gamma_{1}^{A}=\Gamma_{2}^{A}$ in the CMT model. However, during the absorption functionality, the patterned VO_2_ in the 2 A layers are in different states, resulting in *f*_1_ ≠ *f*_2_ and $\Gamma_{1}^{A}\neq \Gamma_{2}^{A}$. Nonetheless, since *d* and *κ* are mainly determined by the original resonant structures, they are almost unaffected when switching between different functionalities. Therefore, by controlling the sheet resistance of the patterned VO_2_ in the 2 A layers, we can control the metasurface to switch between different EM functionalities.

For achieving EM environment sensing, a transparent broadband microstrip antenna and power sensing module are designed. The antenna is integrated on one side of the entire transparent reconfigurable metasurface to enable an integrated design of the actuator and sensor, as shown in Note S2. The specific design process for the power sensing module is shown in Note S3.

### Experiment results

**Fig. 3.**
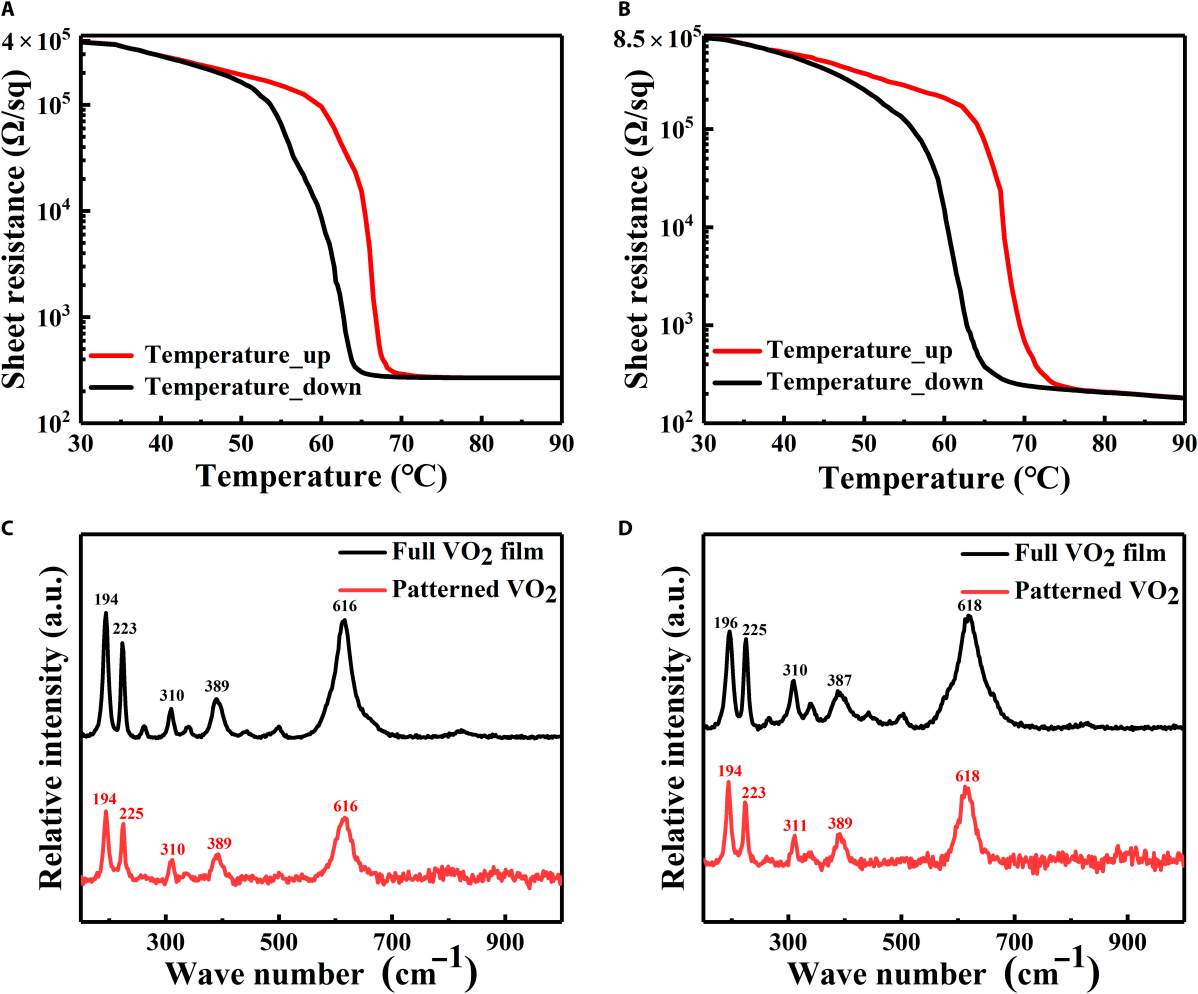
(A and B) Sheet resistance–temperature (*R_sq_* − *T*) measurements of the top and bottom VO_2_ films, respectively. (C and D) Raman spectra measurements of the top and bottom VO_2_ films before and after patterning, respectively.

A metasurface sample was fabricated to verify the broadband high optical transparency and EM manipulation capabilities of the proposed intelligent metasurface and test its optoelectronic performance. Initially, magnetron sputtering coating technology was employed to fabricate the 2 VO_2_ films required for the 2 A layers. The results of the sheet resistance–temperature (*R_sq_* − *T*) test and Raman spectra are shown in Fig. 3. The sheet resistances of the VO_2_ film in the top and bottom layers exhibited a change from 390,000 to 260 Ω/sq and 850,000 to 180 Ω/sq before and after the phase transition, as illustrated in Fig. 3A and B, respectively. The Raman results indicated that the prepared VO_2_ films exhibited a monoclinic phase with reversible phase transition properties. Both layers of the VO_2_ films exhibited the characteristic Raman peaks of VO_2_ before and after patterning, demonstrating that the patterning process did not alter the phase states of VO_2_, as illustrated in Fig. 3C and D. In addition, the x-ray diffraction (XRD) and x-ray photoelectron spectroscopy (XPS) results in Note S4 are used for further characterization.

**Fig. 4.**
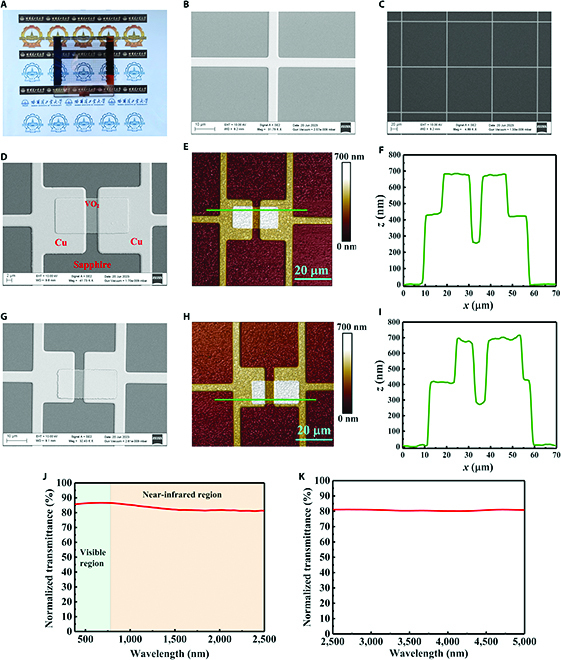
Photographs, SEM, and AFM images, and normalized transmittances of the metasurface sample: (A) physical view of metasurface sample; (B) square metal meshes used for the meshing of the transparent reconfigurable metasurface; (C) radiating layer of the transparent broadband microstrip antenna; (D and E) connections of the top and bottom layers of VO_2_ to the metal meshed resonant structures, respectively; (G and H) connections of the top and bottom layers of VO_2_ to the metal meshed resonant structures, respectively; (F and I) section heights at the green lines in (E) and (H), respectively; and (J and K) normalized transmittances of the metasurface sample from 380 to 2,500 nm and 2,500 to 5,000 nm, respectively.

The final fabricated metasurface sample was 65 × 65 mm and consisted of 20 × 18 transparent reconfigurable metasurface units, as shown in Fig. 4A. The fabricated metasurface sample was very transparent, allowing clear visibility of the university emblem pattern underneath. We first characterized the surface of the sample using confocal microscopy, as shown in Note S5. Then, scanning electron microscopy (SEM) and atomic force microscopy (AFM) were used to clearly observe the structural details of the metasurface sample, as shown in Fig. 4B to I. The SEM characterization results demonstrated that the patterned VO_2_ was well connected to the Cu/Cr interface. The AFM images demonstrated that the thickness of the VO_2_ was approximately 250 nm and the total thickness of the Cu/Cr was approximately 430 nm, which ensured the covering connection of VO_2_ with the metal-meshed resonant structure. As can be seen in Fig. 4G and H, it is a transverse offset of about 3 μm for the patterned VO_2_ in the bottom layer, which is due to the alignment error during the alignment lithography process. In order to analyze whether this offset has any effect on the EM characteristics of the metasurface, we performed CST simulations for different VO_2_ offset methods, as shown in Note S6. The results prove that there is almost no change to the EM characteristics of the entire sample within a VO_2_ offset error of ±5 μm, which also proves that our design has certain process tolerance. Therefore, the offset in Fig. 4G has no effect on the EM characteristics of the sample.

The normalized transmittance was measured within the range of 380 to 5,000 nm to quantitatively analyze the transparency of the metasurface sample, as shown in Fig. 4J and K. The average normalized transmittances in the visible (380 to 780 nm), near-infrared (780 to 2,500 nm), and mid-infrared (2,500 to 5,000 nm) bands were 86.3%, 82.3%, and 80.7%, respectively. This demonstrated the significant transparency of the proposed intelligent EM manipulation metasurface in the visible–infrared band.

**Fig. 5.**
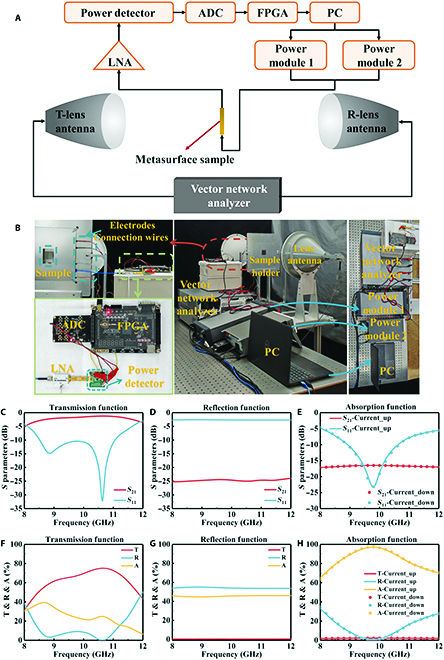
(A and B) Sketch and photograph of the measurement system, respectively. (C to E) Measured *S*-parameters of the transmission, reflection, and absorption functions, respectively. (F to H) Transmittance, reflectance, and absorption calculated using (C) to (E), respectively.

A testing system was set up to further validate the intelligent EM manipulation capabilities of the metasurface, as shown in Fig. 5A and B. The EM function switching capability of the transparent reconfigurable metasurface was verified using 2 power supplies to directly supply the corresponding currents to the 2 A layers. The measurement results are shown in Fig. 5C to H. The patterned VO_2_ films in the 2 A layers were in an insulating state when the bias currents of the 2 A layers were 0 A, as shown in Fig. 5C and F. In addition, the transparent reconfigurable metasurface achieved a passband loss of only 1.24 dB at 10.64 GHz, realizing an EM transmission function with low passband loss. Both patterned VO_2_ films transitioned to a metallic state when the bias currents of the top and bottom A layers were 0.96 and 1.05 A, as shown in Fig. 5D and G, respectively. The metasurface sample blocked the transmission passband and realized over 24 dB of EM shielding within the range of 8 to 12 GHz. Furthermore, its reflectivity was more than 54%, indicating reflection-based EM shielding capability. The bottom patterned VO_2_ was in a metallic state while the top patterned VO_2_ was in an intermediate phase state when the bias current of the bottom A layer remained at 1.05 A and that of the top A layer increased from 0 to 0.75 A or decreased from 0.96 to 0.68 A, as shown in Fig. 5E and G, respectively. The transparent reconfigurable metasurface exceeded 97% high absorption at 9.78 GHz, and absorption rates exceeded 65% within the range of 8 to 12 GHz while the transmittance was nearly zero. This demonstrated that absorption-based EM stealth capability was realized.

**Fig. 6.**
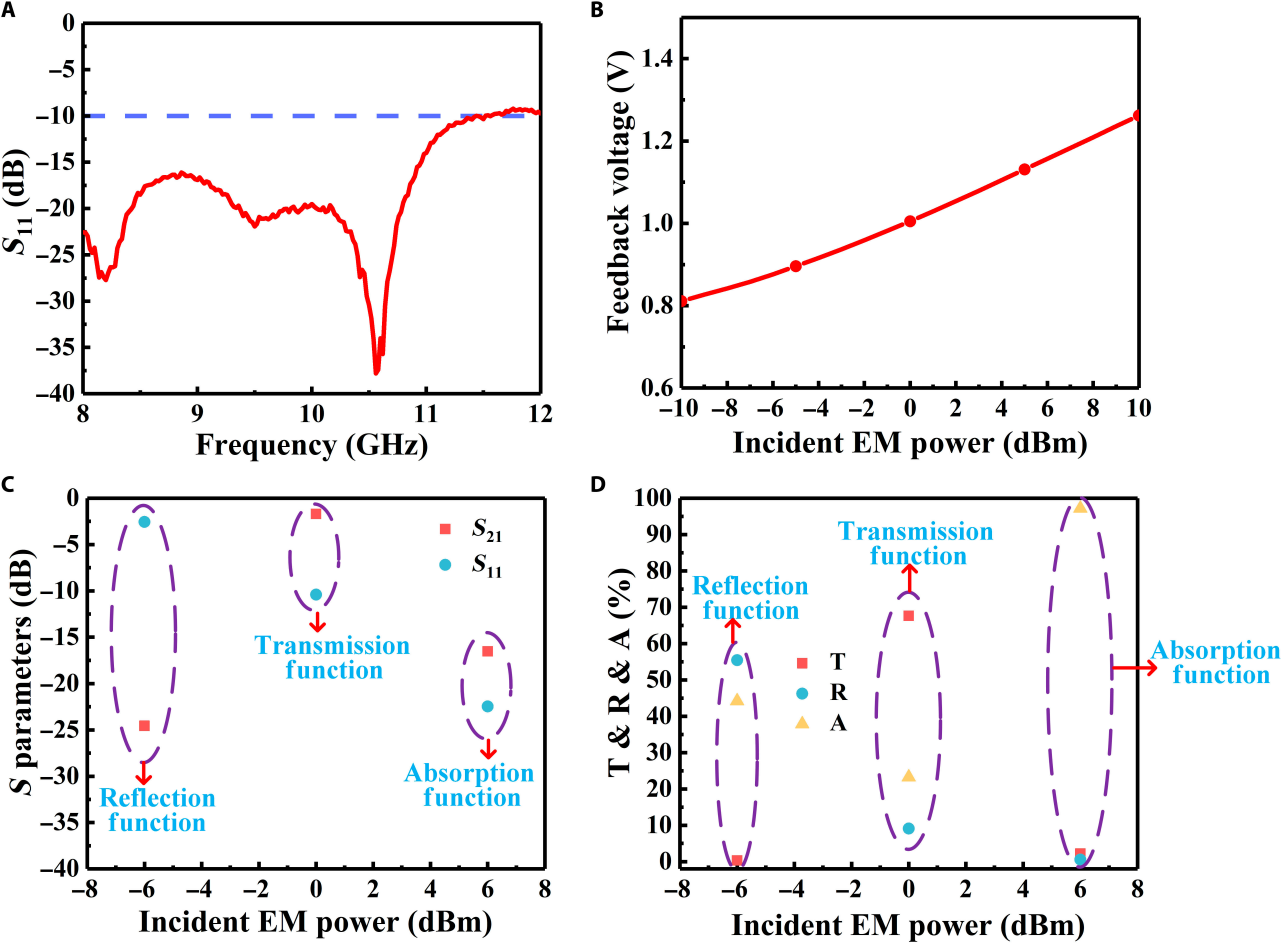
(A) Measured return loss of the integrated transparent broadband microstrip antenna, (B) measured feedback voltage of the power sensing module at 9.78 GHz for different incident power intensities, (C) *S*-parameters at 9.78 GHz for different incident EM wave intensities, and (D) calculated transmittance, reflectance, and absorption at 9.78 GHz for different incident EM wave intensities.

The EM wave-sensing capability of the transparent intelligent EM manipulation metasurface was also tested. The return loss (*S*_11_) of the integrated transparent broadband microstrip antenna in the range of 8 to 12 GHz is shown in Fig. 6A. *S*_11_ remained below −10 dB in the range of 8 to 11.56 GHz, which included the band where the transparent reconfigurable metasurface exhibited transmission loss below 2 dB. The output voltages of the power-sensing module at 9.78 GHz (peak absorption frequency) for different incident powers are shown in Fig. 6B. The incident EM wave power intensity varied within the range of −10 to 10 dBm, thus changing the output voltage of the power sensing module from 0.81 to 1.26 V.

Finally, the bias currents corresponding to the different functionalities of the transparent reconfigurable metasurface were preset in the control program of the host computer to test the intelligent manipulation functionality of the proposed metasurface, as determined from previous tests. The incident wave signal was set as 9.78 GHz. In the control program, EM waves with power < −4 dBm were assumed to be environmental interference waves, those ≥−4 dBm and ≤4 dBm were considered as its own communication waves, and those >4 dBm were considered to be detection waves (*P*_1_ =  −4 dBm, *P*_2_ = 4 dBm). The vector network analyzer was set to continuous wave mode to achieve a single-frequency output at 9.78 GHz. The measurement results were obtained by adjusting the output power of the vector network analyzer, as shown in Fig. 6C and D. The proposed transparent intelligent EM manipulation metasurface identified the incident wave as environmental interference and automatically switched to strong reflection-based EM shielding with an efficiency of 24.57 dB when the incident wave power was −6 dBm (less than −4 dBm). The metasurface recognized its own communication signal and automatically switched to high-transmission functionality with transmission losses of only 1.69 dB when the incident wave power was 0 dBm (greater than −4 dBm and less than 4 dBm). The metasurface determined that it was a possible detection radar wave and automatically switched to strong absorption-based EM stealth capability with 97% absorption when the incident wave power was 6 dBm (greater than 4 dBm). We also performed stability experiments on the sample, and the results are shown in Note S7, which proves that our designed transparent intelligent metasurface has good working stability.

These measurement results demonstrated that the proposed transparent intelligent EM manipulation metasurface not only possessed broadband high transmittance in the visible infrared band but also exhibited the ability to automatically identify the power of EM waves in the environment, enabling adaptive switching to the corresponding EM functionalities.

## Discussion

In summary, a novel intelligent EM manipulation metasurface with broadband high optical transparency was proposed in this study. Patterned VO_2_ were integrated into metal-meshed resonant structures, creating the transparent reconfigurable metasurface, by using the bias current to directly control the phase transition process of the patterned VO_2_ so as to realize different EM functions. Additionally, the designed transparent reconfigurable metasurface was integrated with a transparent broadband microstrip antenna, resulting in an integrated design of the sensor and actuator. Finally, a closed-loop system was constructed in conjunction with the power sensing and feedback control modules. The proposed transparent intelligent EM manipulation metasurface was able to sense the power of environmental EM waves and automatically determine the corresponding EM wave conditions based on preset thresholds. The entire manipulation process to adaptively switch to the corresponding reflection, high transmission, or strong absorption functions was performed automatically without human intervention. Experimental results demonstrated that the proposed metasurface not only possessed intelligent EM regulation capabilities, but also exhibited exceptionally high transmittance of over 80% in the visible–infrared band. These results hold great potential for various applications requiring intelligent EM wave control and high optical transparency.

## Materials and Methods

### Experimental design

First, two 250-nm-thick VO_2_ films were grown on the 65 × 65 × 1 mm and 65 × 65 × 0.5 mm sapphires (crystal orientation 0001) using the magnetron sputtering of a high-purity (99.99%) vanadium metal target in an Ar (99.999%) and O_2_ (99.999%) gas mixture. The distance between the target and substrate was set to approximately 200 mm. The sputtering pressure was set to 0.5 Pa, and the substrate temperature was maintained at 475 °C. After deposition, annealing was conducted at 525 °C in a vacuum furnace at a pressure of approximately 2 Pa. Subsequently, a patterned photoresist layer was created on the VO_2_ film using a laser direct writing system (DWL 66+, Heidelberger Druckmaschinen Aktiengesellschaft) as an etching protection layer. This was followed by etching using reactive ion etching (ME-3A, Institute of Microelectronics, Chinese Academy of Sciences). The etching working power and duration were 100 W and 40 s, respectively. Pure SF_6_ and O_2_ were used as the etching gases at flow rates of 45 and 5 standard cubic centimeters per minute (sccm), respectively. Subsequently, patterned VO_2_ structures were obtained. Next, a second lithography step was employed using an ultraviolet lithography machine (URE-2000S Institute of Photovoltaic Technology, Chinese Academy of Sciences) to fabricate the metal-meshed resonance structures on top of the patterned VO_2_ structures. This was followed by the electron beam evaporation of Cr (10 nm)/Cu (420 nm) and then stripping. Finally, 2 A layers of the metasurface sample were obtained. The specific preparation process is shown in Note S8. The B layer of the metasurface sample was fabricated in the lithography step using an ultraviolet lithography machine.

### Characterization and measurement

The Raman spectra of the VO_2_ film were measured using a micro-Raman spectrometer (Renishaw in Via Reflex) with the laser at a wavelength of 532 nm. The sheet resistances of the VO_2_ films at different temperatures were measured using a baking plate and double-testing digital 4-probe tester (ST2263, Suzhou Jingge Electronic Co., Ltd.). The SEM and AFM images were obtained using SEM (ZEISS Sigma 300) and AFM (BRUKER Dimension Fastscan), respectively. The optical transmittances were measured using an ultraviolet–visible–infrared spectrophotometer (Lambda 1050+, PerkinElmer) in the range of 380 to 2,500 nm, and a Fourier transform infrared spectrometer (BRUKER INVENIO S) in the range of 2,500 to 5,000 nm. XRD pattern is tested by XRD with a Cu Ka1 line (XRD, PANalytical X 'PERT). The x-ray spectrometer used for the test is Nexsa, the ray is Al K Alpha, the full band scan range is 0 to 1,361 eV, and the step size is 1 eV.

The *S*-parameters of the metasurface were measured using a vector network analyzer combined with lens antennas.

## Data Availability

The data used to support the findings of this study are available within the article and the Supplementary Materials. Raw data are available from the corresponding authors upon reasonable request.

## Supplementary Materials

Supplementary 1 Notes S1 to S8 Figs. S1 to S7
