# Supplementary material for: Broadband High Optical Transparent Intelligent Metasurface for Adaptive Electromagnetic Wave Manipulation
Source: Research (Wash D C). 2024 Mar 11;7:0334. doi: 10.34133/research.0334 (PMC10927547; doi:10.34133/research.0334)
Supplement: Supplementary 1 — Notes S1 to S8 Figs. S1 to S7 [file research.0334.f1.zip › supporting information.docx]

**Supplementary Materials for**

**Broadband** **High Optical Transparent Intelligent Metasurface for** **Adaptive Electromagnetic Wave** **Manipulation**

Chao Xia^1,2^, Zhengang Lu^1,2*^, Yilei Zhang^1,2^, Jiubin Tan^1,2^

^1^Ultra-Precision Optical & Electronic Instrument Engineering Center, Harbin Institute of Technology, Harbin 150001, China

^2^Key Lab of Ultra-precision Intelligent Instrumentation (Harbin Institute of Technology), Ministry of Industry and Information Technology, Harbin, 150001, P. R. China

*Address correspondence to: luzhengang@hit.edu.cn

**Supplementary Materials include:**

Supporting Note S1 to S8

Supporting Fig. S1 to S7

**Supporting Note S1**

**The simulation result for B layer**

As shown in Fig. S1, the B layer of the proposed ABA-type metasurface exhibits electromagnetic shielding capability greater than 16 dB from 8 to 12 GHz, which can provide an opaque background for the entire ABA structure in the operating frequency band.

**
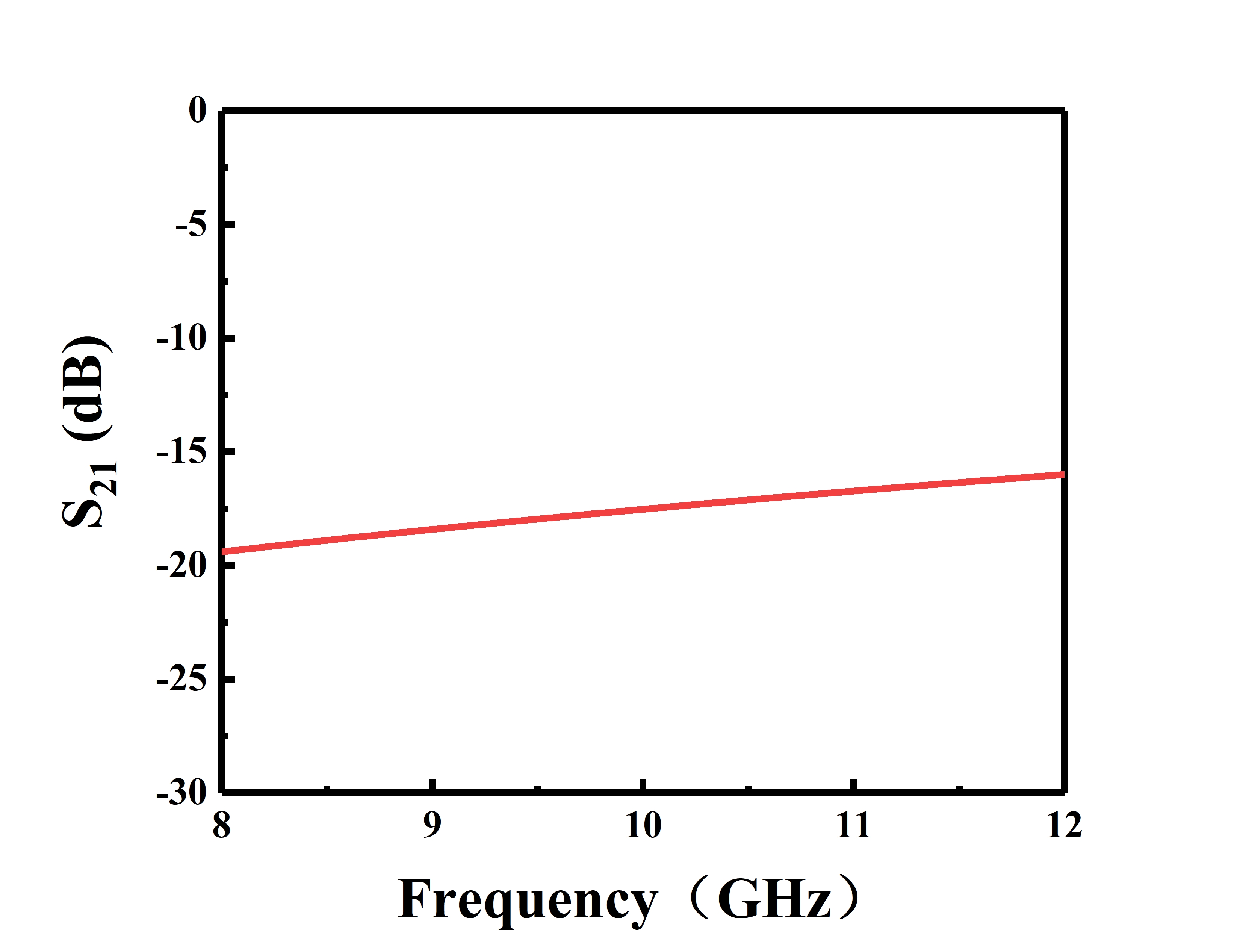
**

**Fig. S1.** The simulation result for B layer of the ABA structure

**Supporting Note S2**

**The design of the transparent broadband microstrip antenna**

As shown in Fig. S2, the designed transparent broadband microstrip antenna consists of a radiating layer and a ground layer located on opposite sides of the substrate of the two A layers. The radiation patch of radiating layer is meshed using metal meshes with a line width of 3$\mu m$ and a period of 150$\mu m$. Due to the metallic feedline portions of the radiating layer and the ground layer need to be connected to the SMA connector, they are not metal-meshed. The structural parameters are $W_{3}=8.8$mm, $L_{3}=6.7$ mm,$W_{4}=3.5$ mm,$L_{4}=0.4$ mm, $W_{5}=2$ mm, $L_{5}=5$ mm, $W_{6}=11$ mm, $L_{6}=4.5$ mm.


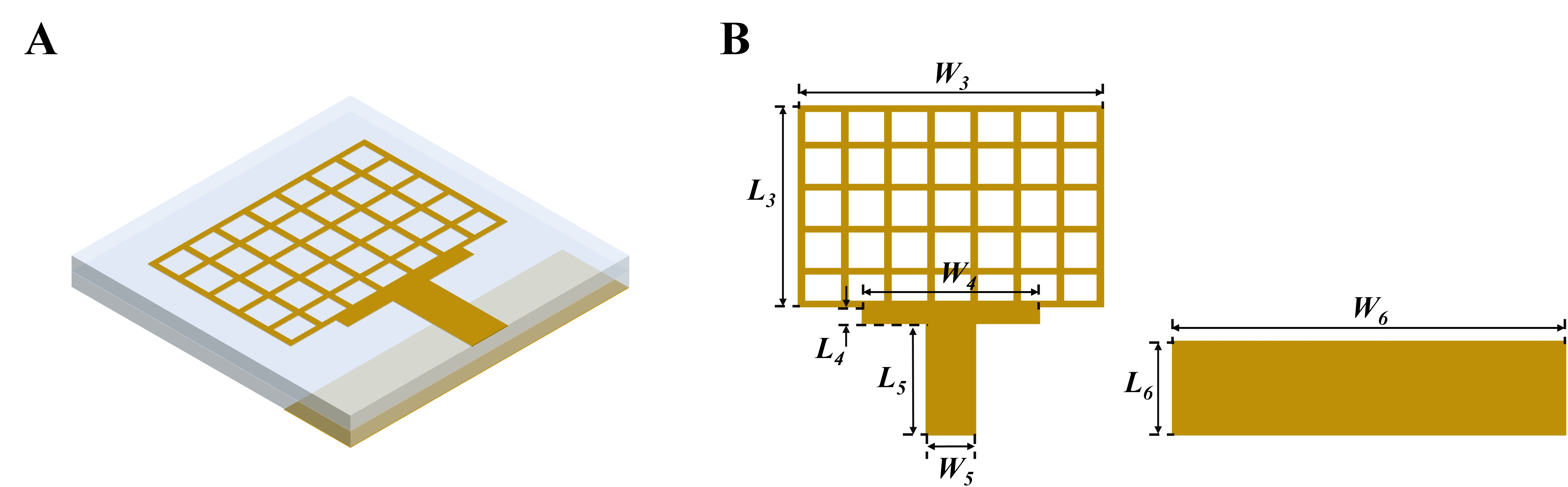


**Fig. S2.** Design of the integrated transparent broadband microstrip antenna. (A) Overall structure of the antenna. (B) Radiating and grounding layers of the antenna.

**Supporting Note S3**

**The design of** **the power sensing module**

Since the power emitted by the vector network analyzer is very low, the transparent broadband microstrip antenna is first connected to a low-noise amplifier through the SMA connector. Then, it is connected to an LTC5582 detector module, and finally, the voltage analog signal output from the detector is converted into a digital signal using an AD7606 module and sent to the FPGA (AX4010). The FPGA receives the voltage digital signal and sends it to the upper computer via a serial port. Due to the hysteresis effect of VO_2_ phase transition, there is also a lag effect during the current-controlled phase transition process, where the current during the descending process is less than that during the descending process for the same VO_2_ sheet resistance state. Therefore, in switching different electromagnetic functions using current-controlled proposed transparent reconfigurable metasurface, according to the simulation results in Fig. 2, the relationship between the bias currents and the corresponding electromagnetic functions should be as follows: for the transmission function, all patterned VO_2_ in the two A layers are in an insulating state, corresponding to no bias current; for the reflection function, all patterned VO_2_ in the two A layers are in a metallic state, and at this time, the two output currents should be at their maximum; while for the absorption function, patterned VO_2_ in the top A layer is in an intermediate state, and patterned VO_2_ in the bottom A layer is in a metallic state, in this case, the bias current for the bottom A layer should be set to its maximum output current, while the bias current for the top A layer needs to consider the lag effect of VO_2_ phase transition when the metasurface switches from reflection to absorption function. Therefore, in the control program, it is necessary to determine whether to increase or decrease the output current based on the current states of the two power supplies and the preset power threshold, and accordingly control the current outputs of Keysight B2910BL and Keysight 2400s for the two A layers.

**Supporting Note S4**

**The XRD pattern and XPS spectra of VO_2_ thin ﬁlm**

As can be seen from the XRD results in Fig.S3(A), the diffraction peak VO_2_(M) (020) is 40.09°. The peak at 41.93°belongs to the diffraction peak of the alumina substrate (0006), beyond that, there are hardly any other impurity peaks in the XRD pattern. The binding energy and oxidation state of VO_2_ were analyzed using XPS spectrum (Fig.S3(B) and(C)). The scanning spectrum of the whole band is shown in Fig.S3(B), where C1s is the surface carbon pollutant, and the rest photoelectron energy lines all point to the V and O elements, indicating that there are no other impurity peaks. Then, we used XPSPEAK peak-splitting software to perform peak-splitting fitting on the obtained curve. The fitting curve is shown in Fig.S3(C) as the red dash line, which basically coincide with the black solid line (the measurement results). The remaining dash lines are peak-splitting results. The binding energy peak at 529.8 eV is the peak at which O^2-^ binds to vanadium ions (O1s). The binding energy peak of V2p_3/2_ is 515.8 eV, which is very consistent with the 516.0 eV of pure VO_2_. V2p_3/2_ shows the V^4+^ and V^5+^ oxidation states, and the V2p_1/2_ peak shows the V^4+^ oxidation state. The area of the V^4+^ oxidation state is much larger than that of the V^5+^ oxidation state, so the prepared VO_2_ films are mainly based on the V^4+^ oxidation state. Combined with XRD and XPS data, it is confirmed that the prepared VO_2_ films are mainly monoclinic VO_2_ (M) at room temperature.


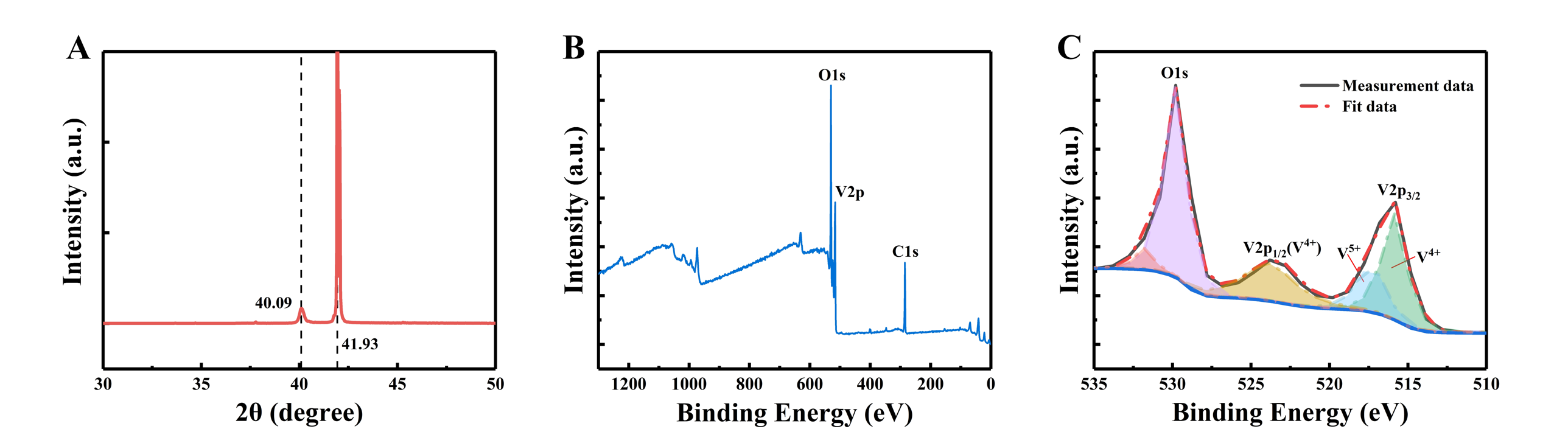


**Fig. S3**. (A) XRD pattern, (B) and (C) XPS spectra of VO_2_ film

**Supporting Note S5**

**The confocal micrograph of the sample**


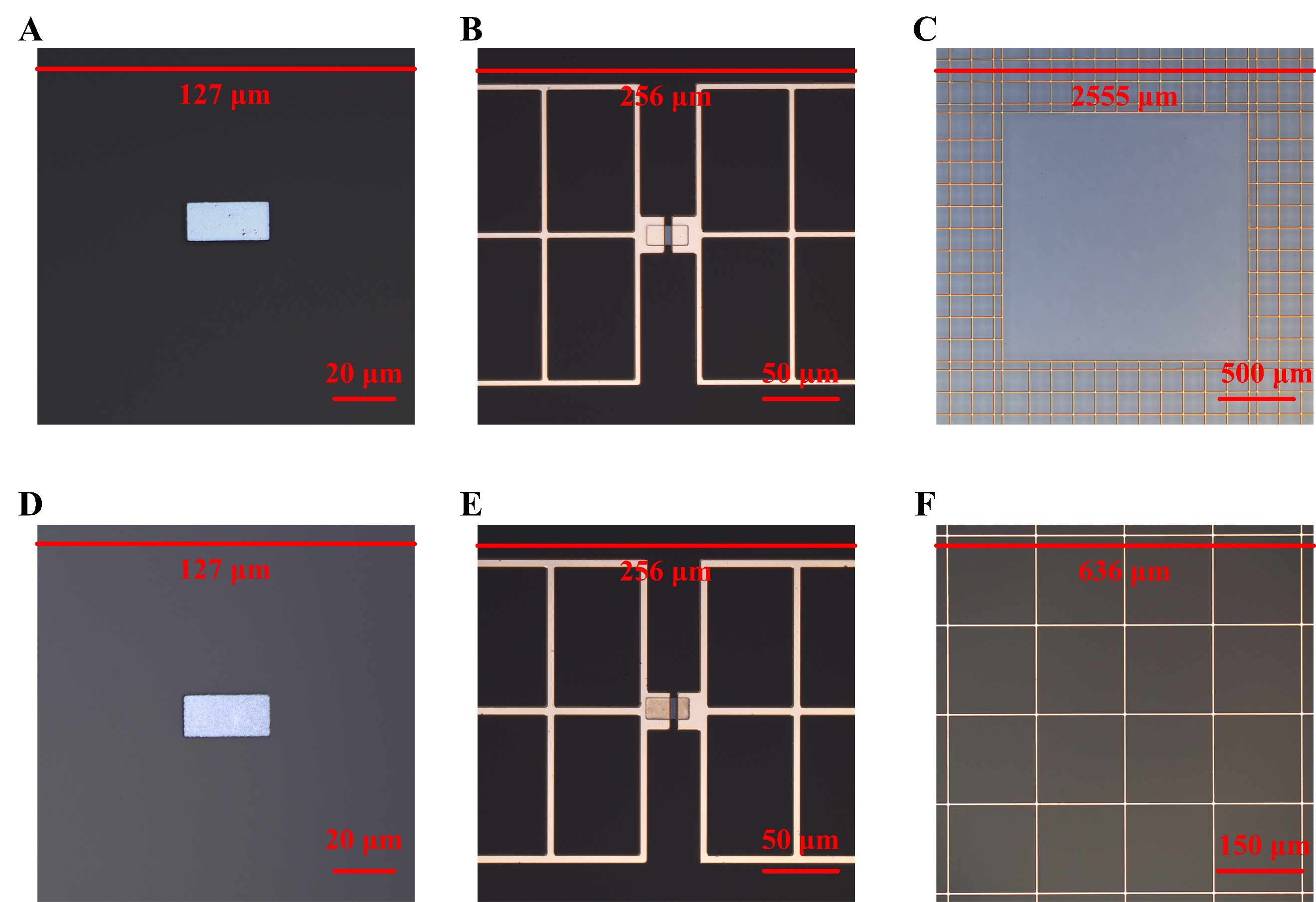


**Fig. S4**. The confocal micrographs of the metasurface sample. (A) Top patterned VO_2_. (B) Connection of patterned VO_2_ and metal meshed resonant structures in the top A layer. (C) B layer. (D) Bottom patterned VO_2_. (E) Connection of patterned VO_2_ and metal meshed resonant structures in the bottom A layer. (F) Square metal mesh used for the meshing of the transparent reconfigurable metasurface.

**Supporting Note S6**

**The** **simulation results for different VO_2_ offset methods**

The VO_2_ offset methods and positions of the top and bottom layers are shown in Figs.S5(A), (B), (C). The offset distances are set to be ±5 μm above and below the off-center position. Figs.S5(D), (G) and (J) show the corresponding simulation results of the offset method in Fig. S5(A). Figs.S5(E), (H) and (K) show the corresponding simulation results of the offset method in Fig. S5(B). Figs.S5(F), (I) and (L) show the corresponding simulation results of the offset method in Fig. S5(C). It can be seen that the simulation results of the three VO_2_ offset methods almost coincide with the no offset results. The results prove that there is almost no change to the EM characteristics of the entire sample within a VO_2_ offset error of ±5 μm, which also proves that our design has certain process tolerance.





**Fig. S5.** The simulation results for different VO_2_ offset methods. (A), (B), (C) are VO_2_ offset methods and positions of the top and bottom layers, the offset distances are set to be ±5 μm above and below the off-center position. (D), (G) and (J) are the corresponding simulation results of the offset method in (A). (E), (H) and (K) are the corresponding simulation results of the offset method in (B). (F), (I) and (L) are the corresponding simulation results of the offset method in (C).

**Supporting Note S7**

**The** **stability experiments of the sample.**

The stability experiment procedures of the sample are as follows:

In the transmission function test, the sample does not need to add bias current. We initially measure the data of group 4, and then after leaving the sample exposed in room temperature for a day, we measured the data of group 5. From Figs.S6 (A) and (E), it can be observed that the data of group 4 and 5 are basically consistent with group 1, demonstrating the good stability of the transmission function for the prepared metasurface at room temperature.

During the reflection function test, bias currents of 0.96 A and 1.05 A were applied to the top and bottom layers, respectively. The data of group 6 was immediately measured. Subsequently, the bias currents were maintained at this level for a 4-hour wait, then, we measured the data of group 7, as shown in Figs.S6 (B) and (E). It is evident that the data of group 6 and 7 are fundamentally consistent with the group 2, demonstrating the reflection function of the prepared metasurface also has good stability at room temperature.

For the absorption function test, bias currents of 0.75 A and 1.05 A were applied to the top and bottom layers, respectively. The data of group 8 was immediately measured, and then the bias currents were maintained at this level. With the increase of time, we found that the absorption of the sample began to deteriorate after about 15 minutes. Analyzing the reason, we found that the overall temperature of the sample increased due to the prolonged flow of current through the sample. Since the top layer of VO_2_ was in the middle of the phase transition process, the increased temperature of the sample caused the top layer of VO_2_ to phase change further and the square resistance to became lower, which made the impedance matching of the sample in the absorption function to be disrupted. So, the absorption of sample was decreased. To solve this problem, we used physical cooling to accelerate heat dissipation (we used an electric fan to blow sideways on the sample), the absorption of the sample was recovered again after about 2 minutes. Then we continued to keep the bias currents and the physical cooling unchanged, and waited for 4 hours before performing the test for the data of group 9, the results are shown in Figs.S6 (C) and (F). The data of group 8 and 9 are basically consistent with the data of the group 3, so the high absorption function of the sample can be maintained for about 15 minutes without physical cooling, whereas it can be stabilized for a long time with physical cooling to accelerate heat dissipation.

The work stably of the transmission and reflection functions is due to the state of VO_2_ is stable. In the transmission function, VO_2_ is a monoclinic phase without phase transition. In the reflection function, VO_2_ is a rutile phase after thorough phase transition. So, the transmission and reflection functions of the sample have good stability at room temperature. In addition, the interval between groups 1-3 and 6-9 was more than two months, indicating that VO_2_ prepared has good stability and current-dependent repeatability.



**Fig.S6**. Stability experiments of electromagnetic transmission properties results. (A) to (C) Measured S-parameters of the transmission, reflection and absorption functions, respectively. (D) to (F) Transmittance, reflectance, and absorption calculated using (A) to (C), respectively.

**Supporting Note S8**

**Preparation process**


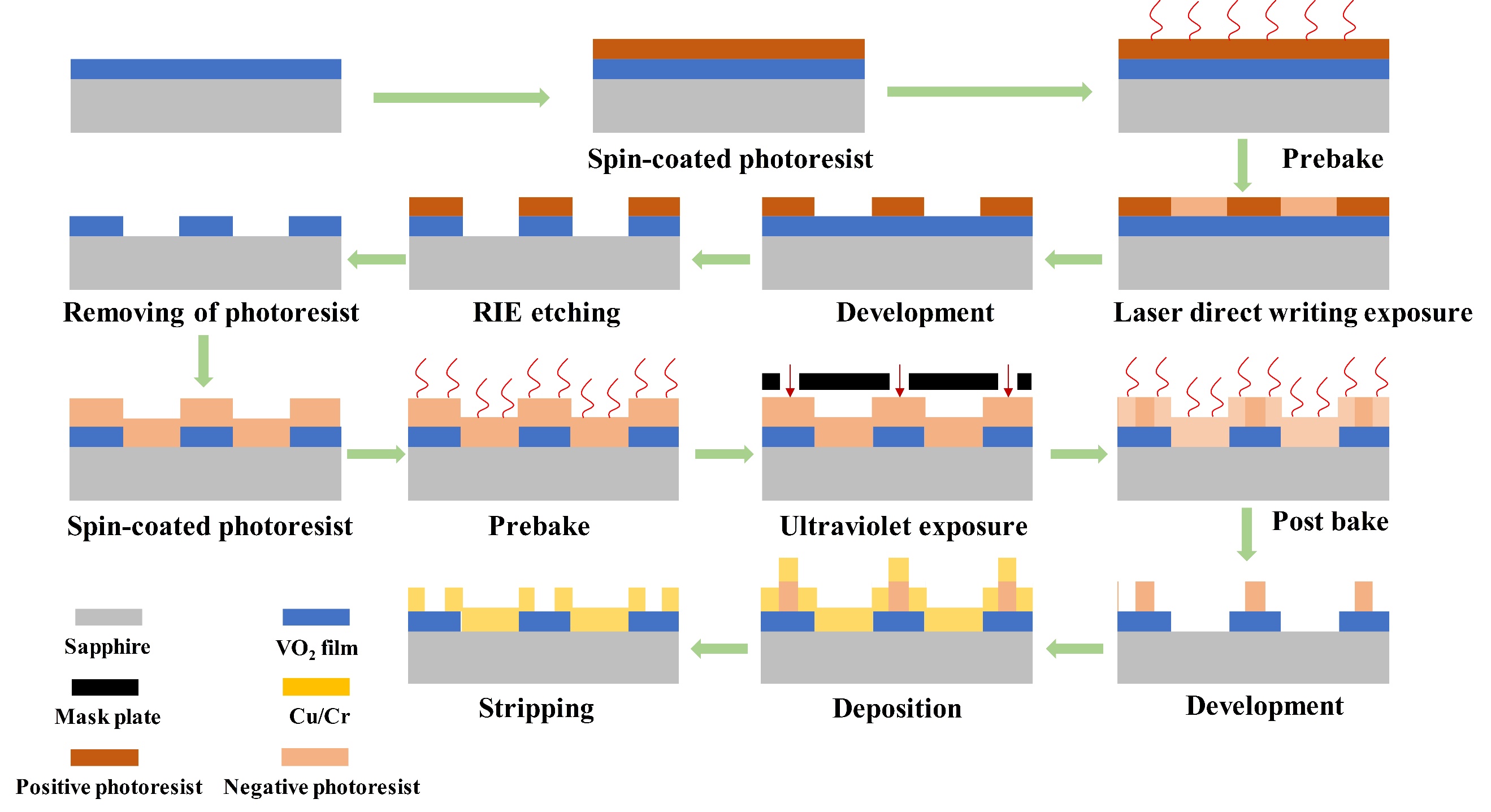


**Fig. S7**. The specific preparation process of the sample.
