# Supplementary figures and images for: Broadband High Optical Transparent Intelligent Metasurface for Adaptive Electromagnetic Wave Manipulation
Source: Research (Wash D C). 2024 Mar 11;7:0334. doi: 10.34133/research.0334 (PMC10927547; doi:10.34133/research.0334)

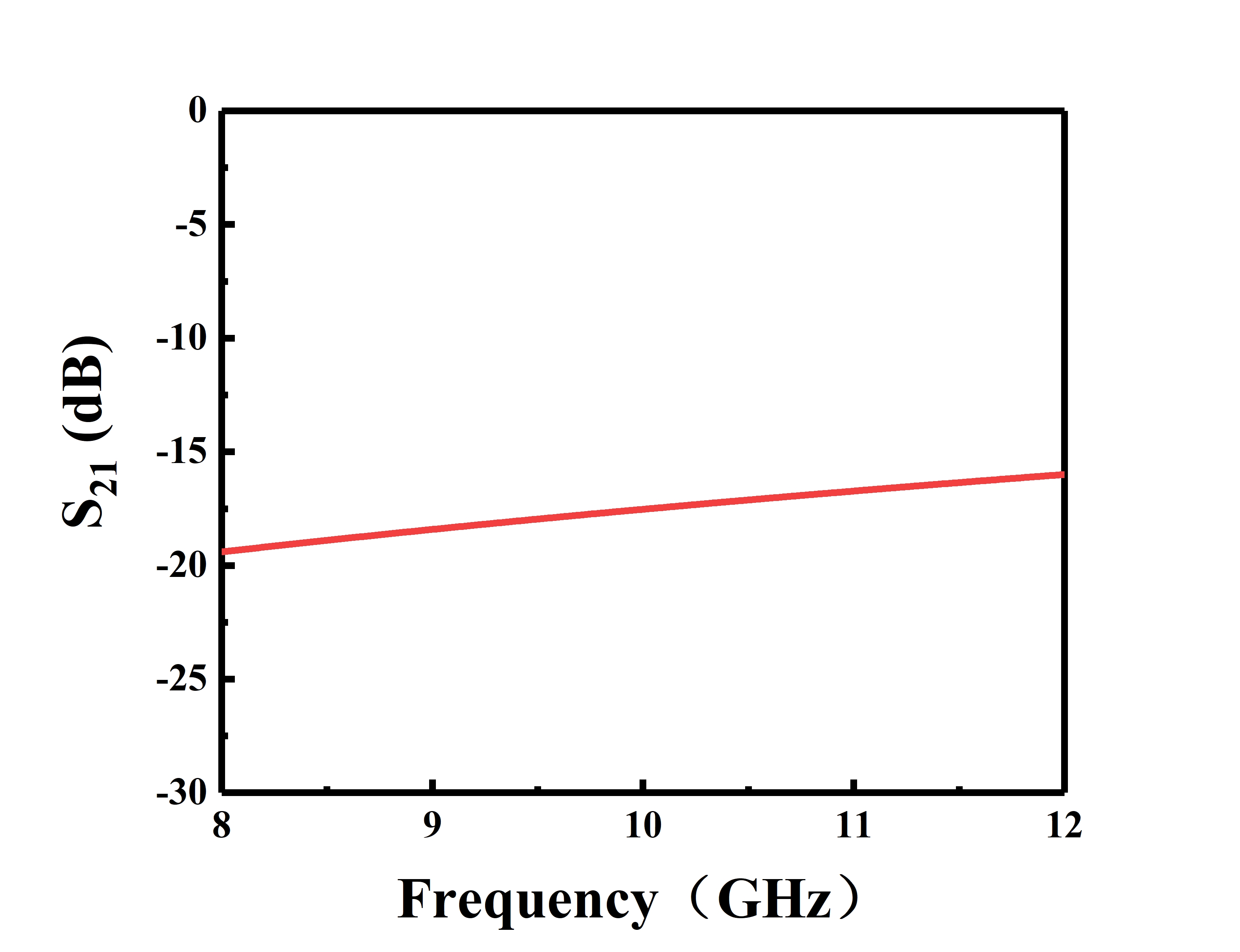

Supplement: Supplementary 1 — Notes S1 to S8 Figs. S1 to S7 [file research.0334.f1.zip › Fig.S1.jpg]

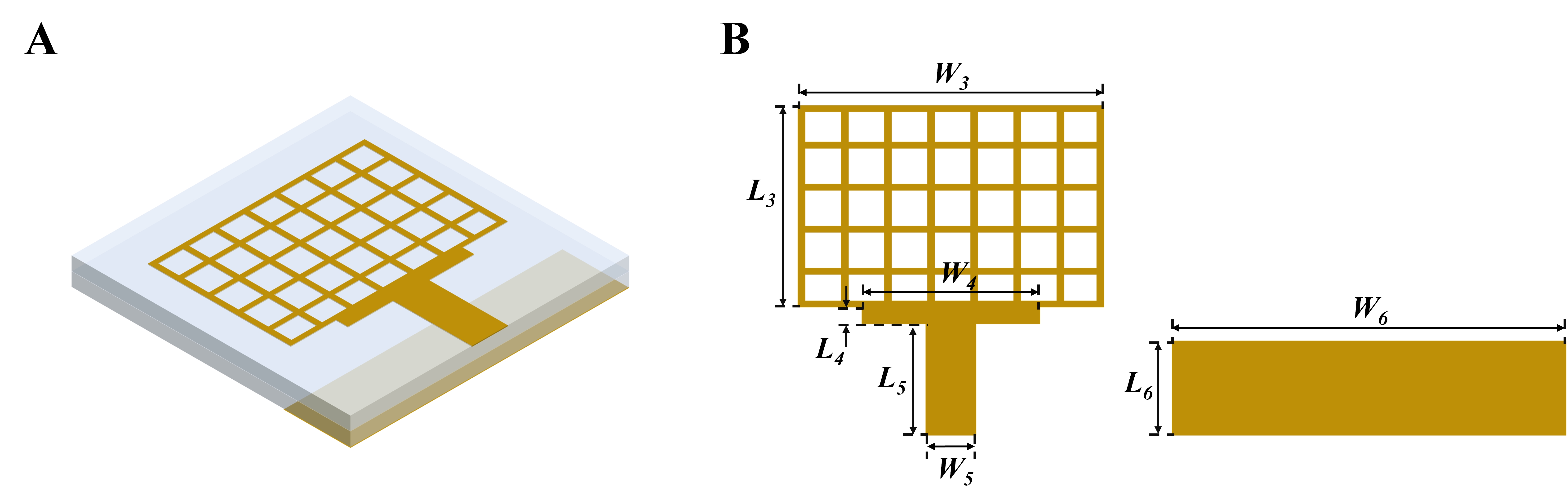

Supplement: Supplementary 1 — Notes S1 to S8 Figs. S1 to S7 [file research.0334.f1.zip › Fig.S2.jpg]

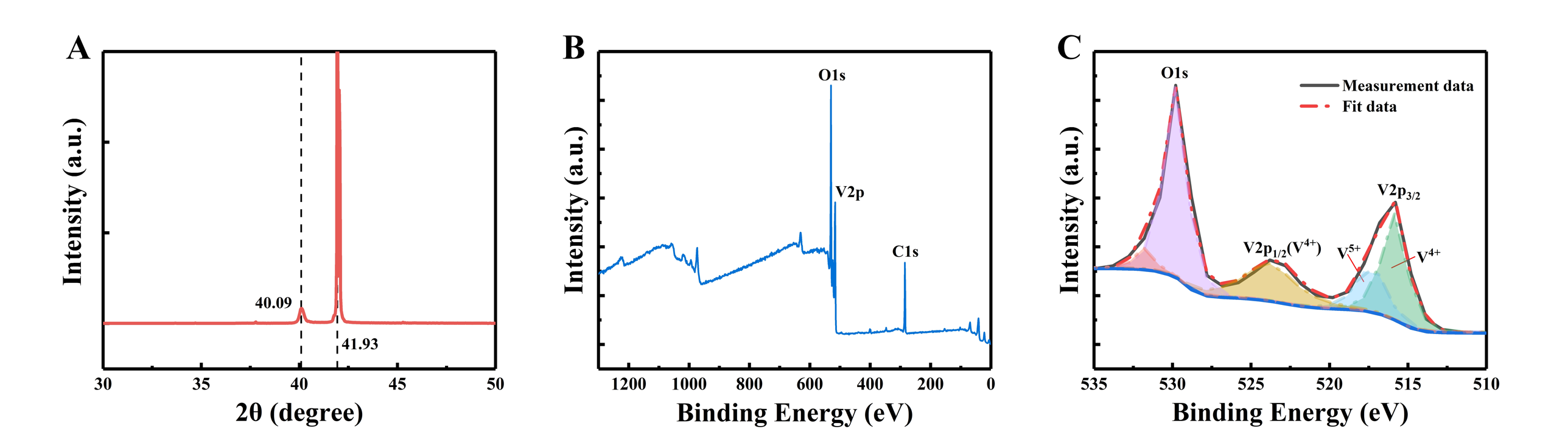

Supplement: Supplementary 1 — Notes S1 to S8 Figs. S1 to S7 [file research.0334.f1.zip › Fig.S3.jpg]
